# Cardiomyopathy Associated With Coronary Arteriosclerosis in Free-Ranging Eurasian Lynx (*Lynx lynx carpathicus*)

**DOI:** 10.3389/fvets.2020.594952

**Published:** 2020-12-21

**Authors:** Marie-Pierre Ryser-Degiorgis, Nadia Robert, Roman Kaspar Meier, Samoa Zürcher-Giovannini, Mirjam Pewsner, Andreas Ryser, Urs Breitenmoser, Alan Kovacevic, Francesco C. Origgi

**Affiliations:** ^1^Centre for Fish and Wildlife Health, Vetsuisse Faculty, University of Bern, Bern, Switzerland; ^2^Foundation KORA, Bern, Switzerland; ^3^Small Animal Clinic, Department of Clinical Veterinary Science, Vetsuisse Faculty, University of Bern, Bern, Switzerland

**Keywords:** cardiomegaly, heart failure, heart murmur, lung edema, myocardial fibrosis, pathology, wildlife

## Abstract

The Eurasian lynx (subspecies *Lynx lynx carpathicus*) was reintroduced to Switzerland in the 1970's. Health monitoring of the reintroduced population started in the late 1980's. Since then, six lynx have been found affected by a myocardial disease. The earliest case was an animal that died after a field anesthesia. Two lynx were found dead, two were euthanized/culled because of disease signs, and one was hit by car. Two had a heart murmur at clinical examination. At necropsy, the first animal showed only lung edema but the other five had cardiomegaly associated with myocardial fibrosis. Three had multisystemic effusions. Histological examination of all six lynx showed mild to severe, multifocal, myocardial interstitial and perivascular fibrosis along with multifocal myocyte degeneration and loss, and replacement fibrosis. Moderate to severe multifocal arteriosclerosis with associated luminal stenosis of the small and medium-sized intramural coronary arteries and the presence of Anitschkow cells was also observed. The heart lesions may have led to sudden death in the first case and to a chronic right-sided heart failure in the remaining. None of the lynx showed lesions or signs suggestive of an acute or subacute infection. Given the common geographic origin of these animals and the severe loss of heterozygocity in this population, a genetic origin of the disease is hypothesized.

## Introduction

Cardiovascular disease is considered the leading cause of mortality in people worldwide (1). Spontaneous cardiomyopathies have been described in multiple domestic animals such as dogs, cats, cattle and pigs (2–4), as well as in a wide range of wild animal species, from reptiles to non-human primates (5–10). Among felids, the domestic cat is known to be particularly prone to develop cardiomyopathies, and it was proposed as a model for human disease (11). By contrast, descriptions of cardiac muscle disorders in wild felids are rare (12).

In both domestic animals and humans, the term cardiomyopathy has been long used to refer to idiopathic myocardial disorders, but cardiomyopathies were subsequently defined as clinicopathological entities resulting either from genetic anomalies or from nutritional deficiencies, with cardiomegaly and myocardial fibrosis as most typical features (13). Meanwhile, definitions have evolved and, based on the literal meaning of the word (heart muscle disease), the American Heart Association has proposed a classification system including primary (heart disease only) and secondary (systemic disease with cardiac involvement) cardiomyopathies (14). Most recently, veterinary cardiologists have published a consensus document on the classification of cardiomyopathies in cats, in which they define cardiomyopathy as a “myocardial disorder in which the heart muscle is structurally and functionally abnormal in the absence of any other cardiovascular disease sufficient to cause the observed myocardial anomaly.” They recommend the further use of the formerly described four main forms of cardiomyopathies but to consider them different disease phenotypes rather than different disease forms (15): (1) dilated (congestive) cardiomyopathy (DCM), characterized by enlargement and dilation of both atria and ventricles with normal or reduced ventricular wall thickness; (2) hypertrophic cardiomyopathy (HCM), typically featured by a wide phenotypic variability of left ventricular hypertrophy with reduced lumen size, increased absolute and relative heart weight, hypertrophic papillary muscles and stiff wall. Atrial dilation, right ventricular hypertrophy, pulmonary edema and pleural effusion may be present. Myocyte disarray is the gold standard for histopathological HCM diagnosis but variable degrees of coronary arteriosclerosis and replacement fibrosis are also common; (3) restrictive cardiomyopathy (RCM), characterized by bilateral atrial dilation with ventricles of normal size with a prominent bridging endocardial scar within the left ventricle; (4) arrhythmogenic right ventricular cardiomyopathy (ARVC), characterized by a severe right atrial and ventricular dilation, frequently with right ventricular wall thinning. Cases with overlapping features or not fitting well into any category can be referred to as unclassified cardiomyopathies or as cardiomyopathies with a non-specified phenotype (13, 15–17).

The most common type of cardiomyopathy and frequent cause of morbidity and mortality in domestic cats is HCM. This disease concerns mainly older cats and is often associated with a loud systolic heart murmur. Although it frequently remains subclinical, a majority of affected cats develop congestive heart failure, others arterial thromboembolism, and a minority die unexpectedly in absence of clinical signs (15, 18–20). In humans, the definition of cardiomyopathy formerly excluded coronary arterial or other vascular disorders (13). Nevertheless, inherited HCM was shown to be associated with an increased wall/lumen ratio in small arterioles, predisposing patients to ischemia and subsequent myocardial fibrosis, with these changes evolving for years before the onset of symptoms (21). Similarly to cats, disease is often subclinical, and possible outcomes include sudden death, which typically occurs in apparently healthy young patients during physical exercise, thromboembolic events, and chronic heart failure (22).

The Eurasian lynx (*Lynx lynx*) is a wild felid endemic to Europe and North-central Asia, which has undergone a massive population reduction in Europe in the 19th century (23). Despite major conservation efforts including reintroduction programs, many local populations remain small and fragile, and human persecution together with other anthropogenic factors continue to pose a serious threat to the long-term survival of this large carnivore (23–25). Furthermore, only a very limited number of individuals were released in all reintroduction projects, causing severe genetic bottlenecks that may have yet unknown impacts on the fitness of the reintroduced populations (26, 27). In this context, knowledge of causes of mortality and emerging diseases of infectious and non-infectious origin in Eurasian lynx, in relation to population size and status, is essential.

The Carpathian lynx (*L. lynx carpathicus*), which currently only occurs in small fragmented populations in Western, Central and Eastern Europe, is one of the nine currently proposed Eurasian lynx subspecies (28), of which three others occur in Europe: the Northern or Boreal lynx (*L. lynx lynx*) in Scandinavia, Finland and European Russia, the Balkan lynx (*L. lynx martinoi*) in the Balkan region and the Caucasian lynx (*L. lynx dinniki*) in Turkey. Although the separation into these subspecies remains controversial (29, 30) and genetic differences among subspecies and populations may be due to epigenetic changes driven by local environmental characteristics, the existence of differences are undisputable (27, 28) and associated with varying phenotype characteristics, such as body size and weight, coat pattern and skull shape, as well as varying life history traits (28, 31, 32).

Here we report previously undescribed cardiac disorders characterized by myocardial fibrosis and intramural coronary arteriosclerosis in six free-ranging Carpathian lynx from a reintroduced population in Switzerland.

## Materials and Methods

All lynx included in this study were free-ranging dead animals submitted to the Centre for Fish and Wildlife Health of the University of Bern (FIWI) for post-mortem examination in the framework of the lynx health monitoring program in Switzerland (33). Accordingly, no ethical permit was required for the post-mortem study. Four lynx died spontaneously in nature. One was culled by a professional state game warden in accordance with the Swiss federal law on hunting practices and protection of free-ranging wild mammals and birds (nr. 922.0). Another lynx was euthanized by a veterinarian because of severe debilitation. Capture and marking procedures were performed with authorizations obtained in the framework of ecological projects (34–36). The presence of a veterinarian at wildlife captures is not required by the Swiss legislation and the inclusion of a wildlife veterinarian in the lynx capture team dates back to 2000, meaning that only case 3 and 5 underwent clinical examination. However, no veterinarian participated in the last capture of case 3, for which a video and clinical records were made by trained biologists and the records subsequently evaluated by a veterinarian.

The lynx population in the northwestern Swiss Alps originated from probably <10 of the 26 individuals released in Switzerland in the 1970's (28, 37). The local population in the northwestern part of the Swiss Alps is isolated and presently consists of 50–70 independent lynx (38). According to the Swiss Lynx Management Plan, all lynx found dead or culled because of compromised health must be submitted to the Centre for Fish and Wildlife Health (FIWI) at the University of Bern for an in-depth, standardized post-mortem examination following a protocol in place since 2002 (33, 39).

Six lynx older than 1 year and of both sexes submitted for necropsy to the FIWI between 1988 and 2014 were included in this study. The four most recent cases were examined by the authors and documented with photographs. The two earliest cases were retrieved from the archives. Selection criterion was the record of information suggestive of cardiomyopathy in the case history, macroscopic pathologic observations and/or histological descriptions. Lynx were aged as accurately as possible by cementum annuli counts, tooth wear evaluation and body measurements, and placed into age classes as previously described (40, 41).

**Figure 1:**
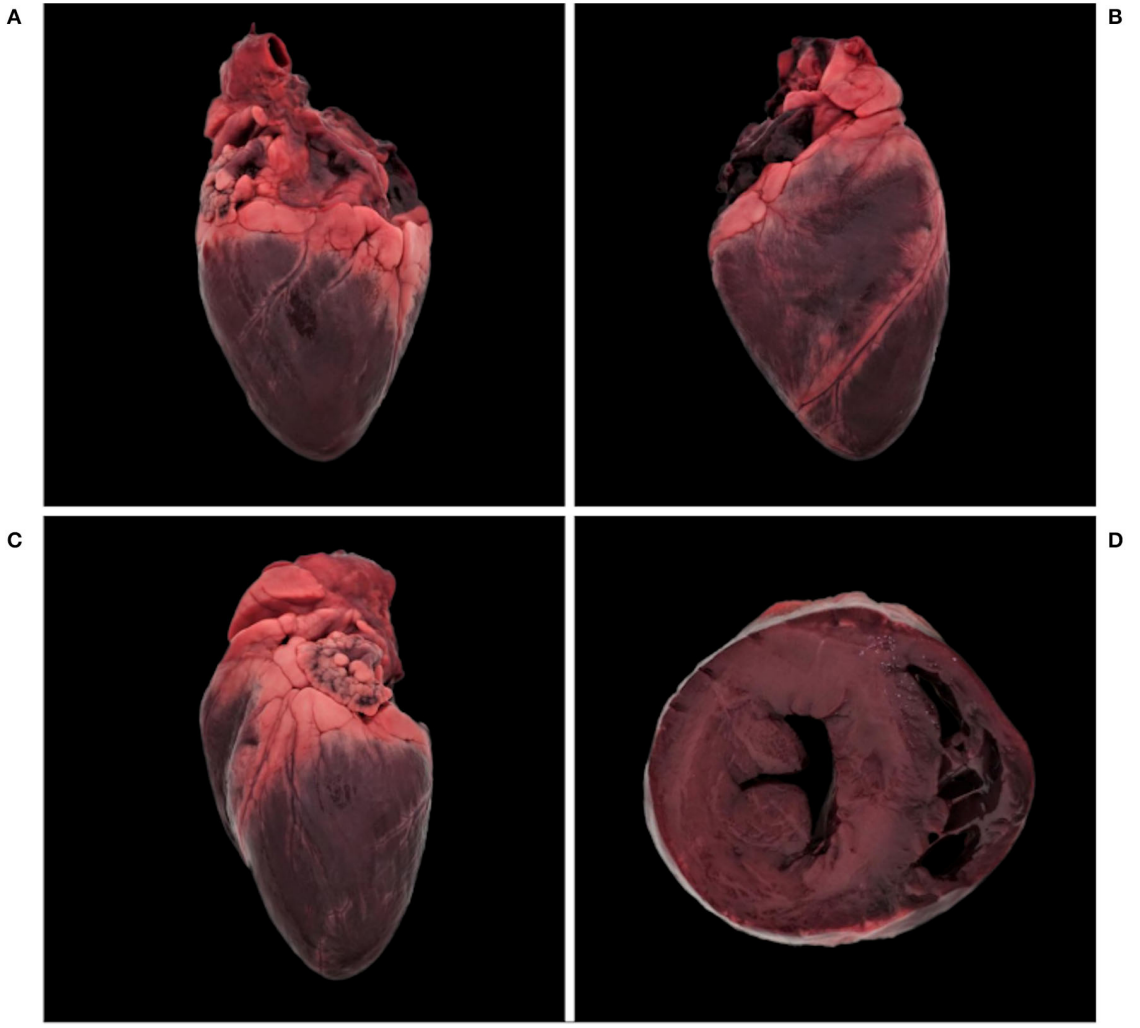
Normal heart, Eurasian lynx (*Lynx lynx carpathicus*); **(A)** right lateral view, **(B)** diaphragmatic view, **(C)** left lateral view, and **(D)** cross-section. Dark coloration is a freezing artifact.

Since there are no published reference values for the morphology of the Carpathian lynx heart, and those generated for Northern lynx do not include the ratios necessary to overcome body size and weight differences (28, 42), a healthy individual from Switzerland, originally taken into a rehabilitation station as an orphan and roadkilled after its release, was used as a “control” animal for comparison (Table 1, Figure 1). Repeated auscultation performed under anesthesia with medetomidine and ketamin (39) did not reveal any anomaly. Findings were recorded using an electronic stethoscope (3M^TM^Littmann^®^ 3200) and later confirmed by a board-certified cardiologist. Physical examination, hematology and blood chemistry, radiographs of the thorax and echocardiography in clinical settings, as well as pathological examination after traumatic death (necropsy and histology) were also consistent with a healthy heart.

**Table 1:** Semiquantitative scoring scheme for arteriosclerosis, myocardial fibrosis, and Anitschkow cell presence in histological sections of the heart of six Eurasian lynx (subspecies *Lynx lynx carpathicus*) from Switzerland affected by cardiomyopathy.

**Feature**	**Grade**	**Definition**
Arteriosclerosis^a^	1	≤ 5 vessels mildly to moderately affected
	2	≤ 5 vessels moderately to severely affected, or > 5 vessels but < 50% of the vessels mildly to moderately affected
	3	> 50% of the vessels mildly to severely affected
Myocardial fibrosis	1	Thin strands of collagen expanding between myocytes
	2	Thicker collagen bands up to small areas of replacement fibrosis
	3	Large amounts of collagen deposits, including large areas of replacement fibrosis
Anitschkow cells^b^	1	≤ 5
	2	6–15
	3	> 15

a *If there was a marked variability among the available sections, intermediate grades were given*.

b *Number of cells per HPF in an area with obvious tissue changes*.

For the five most recent cases and the control, morphological heart data recorded post-mortem included heart weight (g) and ventricular wall thickness (mm). The right ventricular free wall, interventricular septum and left ventricular free wall were measured on a transverse cross section (perpendicular to the long axis of the ventricles) from the epicardium to the endocardium, excluding papillary muscles, as a single measurement, using a millimeter rule. This procedure was the same as reported in domestic cats (16) except that for lynx the measurements were made on a cross section located half instead of a third of the distance from the apex to the base, similarly to a study comparing cats and rhesus macaques (*Macaca mulatta*) (43).

Representative samples of the lung, heart, kidneys (case 1–6, control); liver, spleen, genital organs (case 2–6, control); stomach, intestine (case 3–6, control), adrenals (case 3–6); lymph nodes (case 3, 4, and 6); skeletal muscle (case 3–5, control); pancreas (case 2 and 4); thyroid (case 4 and 5); urinary bladder (case 4 and 6, control); and brain (case 5) were collected and fixed in 10% neutral-buffered formalin. Tissues were then embedded in paraffin, sectioned at 5 μm and stained with hematoxylin and eosin (H&E) for histologic examination following the accredited protocols of the Institute of Animal Pathology, Vetsuisse Faculty, University of Bern. Archived paraffin blocks and/or histological sections were available for case 2–6. Additional heart sections were stained using the Masson's trichrome (MT) and Van Gieson (VG) protocols for a qualitative and semi-quantitative evaluation of collagen proliferation and vascular changes.

Histological assessment of the available heart sections was performed with board-certified pathologists, focusing on the left free ventricle wall and septum, which were shown to be more severely affected than the right free ventricle wall in a preliminary retrospective histological study (44). The two main pathological features (arteriosclerosis and myocardial fibrosis) were graded separately according to an arbitrarily defined set of features. The grading for arteriosclerosis took both the number of affected vessels and the severity of the arterial wall thickening into account. Changes were classified based on their severity and ranged from mild (minor expansion of the media and/or subintimal collagen deposits) to severe (obvious luminal stenosis). Myocardial fibrosis was either perivascular, interstitial, or variably extensive with replacement of myocytes, or combinations of these. Like for arteriosclerosis, grading of the myocardial fibrotic changes was based on subjective assessment of the amount of collagen deposits. The number of Anitshkow cells per high power field was counted in an area with obvious tissue changes (Table 1).

## Results

### Animal Demographics and Clinical History

General information on the six cases and the control are given in Table 2. All cases came from the same geographical area in the northwestern part of the Swiss Alps, while the control was found in the Jura Mountains, a separate population across the French/Swiss border. Case 1 and 2 and the control were ≤2 years old while the other four lynx were ≥7 years old. All were males except for case 2.

**Table 2:** General information on six Eurasian lynx (subspecies *Lynx lynx carpathicus*) from Switzerland affected by cardiomyopathy and one healthy individual (control) for comparison.

**Parameters**	**Case 1**	**Case 2**	**Case 3**	**Case 4**	**Case 5**	**Case 6**	**Control**
Year of death	1988	1991	2003	2012	2013	2014	2015
Circumstances of death	Found dead	Found dead	Found dead	Culled	Euthanized	Hit by car	Hit by car
Captured at least once	Yes	No	Yes	Yes	Yes	No	Yes
Sex	Male	Female	Male	Male	Male	Male	Male
Age class (age in years)	Adult (≥3)	Subadult^a^ (1)	Adult (7)	Adult (14)	Adult (8)	Adult (8)	Subadult (1.5)
Body weight (kg)	20.5	21	20.1	19.3	15.3	24.8	18.9
Corrected BW (kg)	n/a	12	16.7	16.1	n/a	n/a	n/a
Body condition	Excellent	Cachexia	Cachexia	Moderate emaciation	Severe emaciation	Excellent	Good

*BW, body weight; n/a, not available. Corrected body weight was obtained after subtracting 1 kg per liter of free fluid (including an approximative amount for subcutaneous edema)*.

a *The weight was clearly in the adult category (41) and histological examination of the ovaries revealed corpora lutea and follicles in several stages of development (sexually mature animal) but tooth cementum annuli analysis revealed an age of 17 months (subadult age class)*.

Case 1 was found dead after a field anesthesia with xylazine and ketamine (34) for fitting a radio-collar in June 1988. This lynx was apparently healthy but unexpectedly found dead the next day at the location where it had been left for recovery, suggesting that it died because of an anesthesia accident shortly after the manipulations.

Case 2 was found dead. The original diagnosis of feline infectious peritonitis was not confirmed by immunohistochemistry (45) and the cause of death remained unclear. Case 3, 4, and 5 were first anesthetized in the field as clinically healthy lynx using medetomidine and ketamine (39) and fitted with a VHF radio-collar (very high frequency or pulse collar; A. Wagener, Köln, Germany).

Case 3, first captured in September 1999, was recaptured twice (2 and 4 years after the first capture). At the second capture in February 2001 it still appeared healthy and was transported to a quarantine station to be subsequently translocated to northeastern Switzerland (39, 46). It was in good body condition (23 kg) and physical examination at capture, blood analyses and regular direct observations during 2 weeks of quarantine did not reveal overt sign of illness. During the pre-release clinical check in March 2001, a soft heart murmur was detected but all other evaluated health parameters were inconspicuous, including peripheral pulse, heart rate (56–72 beats/min.), color of oral mucosa, and capillary refill time (<3 s). The lynx was released into the wild equipped with a new VHF collar. Radiotelemetry indicated that it showed a normal, species-specific spatial and hunting behavior until 15 April 2003, when it was trapped again and anesthetized with the same protocol to replace its radio-collar. It showed an abnormally long induction and recovery times, an elevated heart rate (100–117 beats/min.), and a delayed capillary refill time (≥3 s) although mucosal color remained inconspicuous. A video record of the lynx approaching the trap revealed a severely distended, possibly oscillating abdomen suggestive of ascites. Considering the heart murmur noticed 2 years earlier, a presumptive diagnosis of cardiac circulatory insufficiency was made. It recovered well from the anesthesia, but within 3 weeks after this last capture its health status progressively deteriorated (abnormal spatial and feeding behavior after 7 May 2003) until death occurred (found dead on 20 May 2003).

Case 4 was caught in February 1998 and had shown a normal post-capture spatial and hunting behavior until its radio-collar stopped working early March 2001. In February 2012 (14 years after capture) it was found lying close to a road without escape reaction and therefore assumed to be ill and culled.

Case 5 was in good body condition (24 kg) when captured mid-March 2013, and all recorded health and anesthesia parameters were normal except for a heart murmur. It was re-captured and euthanized 7 weeks later because of abnormal behavior (apathy and ataxia) and severe emaciation. It developed respiratory insufficiency during transport but could be stabilized with an intramuscular injection of 40 mg Doxapram (Dopram^®^, Chassot AG, Switzerland) and arrived still alive at the University of Bern, where it was euthanized. Heart auscultation prior to euthanasia confirmed the presence of a loud systolic heart murmur nearly completely covering the heart sounds.

Case 6 was killed in a traffic accident.

### Pathology

**Figure 2:**
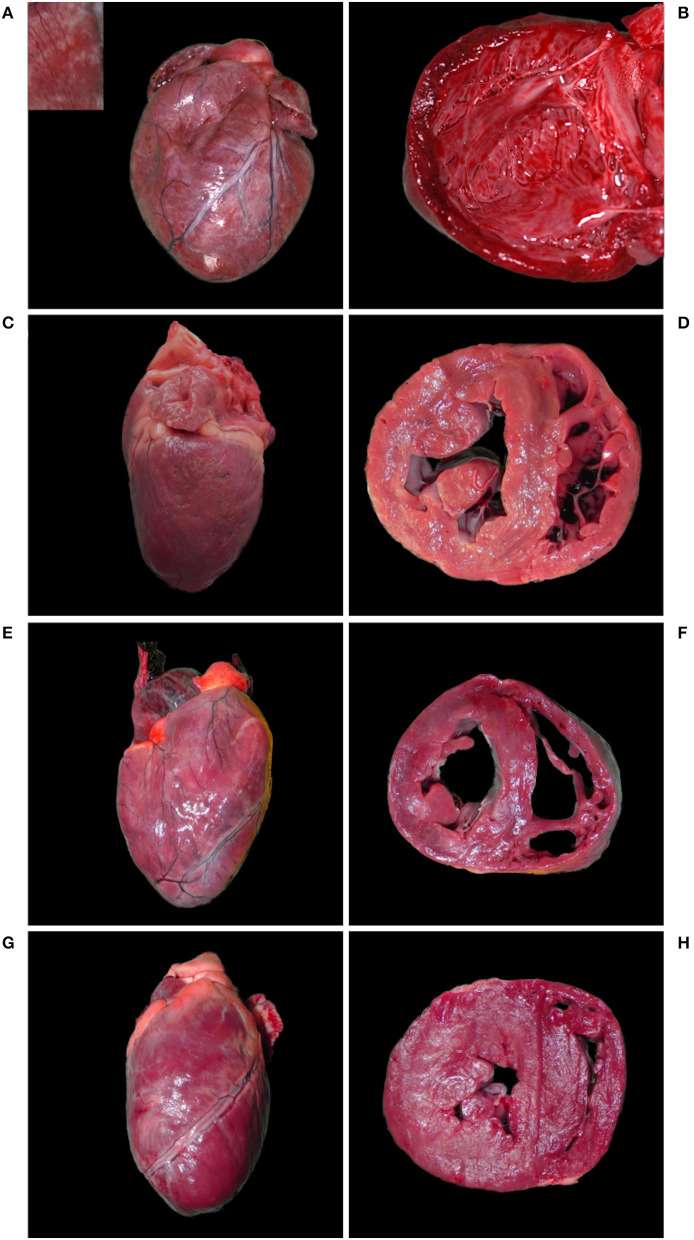
Hearts with cardiomegaly and myocardial fibrosis, Eurasian lynx (*Lynx lynx carpathicus*); **(A)** case 3, diaphragmatic view: severe cardiomegaly with rounded profile and marked multifocal fine coalescent light tan striations running across the surface (inset) in absence of coronary fat deposits; **(B)** case 3, left ventricle: severe dilation with attenuation of wall thickness and papillary muscles; **(C)** case 4, left latero-diaphragmatic view: pale color and moderate cardiomegaly, uneven surface with variably extensive minimally to mildly depressed areas and discrete multifocal light tan foci (inset); **(D)** case 4, cross-section: moderately expanded left ventricle lumen; **(E)** case 5, diaphragmatic view: moderate cardiomegaly with relatively normal profile and multifocal to coalescent light tan foci; **(F)** case 5, cross section: severe dilation of both ventricles; **(G)** case 6, diaphragmatic view: moderate cardiomegaly with elongated ovoid shape, lateral bulging of the right ventricle, multifocal to coalescent light tan foci and uneven surface; **(H)** case 6, cross-section: bilateral ventricular hypertrophy.

Case 1, a young individual in excellent body condition, only showed a severe, acute, bronchoalveolar edema and emphysema, and moderate fibrosis of the mitral valve. The other five cases (case 2–6) had cardiomegaly together with multifocal fine light tan striations coalescing to prominent tan foci in the myocardium (multifocal myocardial fibrosis). Cardiomegaly was associated with marked dilation of either the right (case 2) or left ventricle (case 4), or both (case 3 and 5), while bilateral ventricular hypertrophy was present in case 6 (Figure 2).

**Figure 3:**
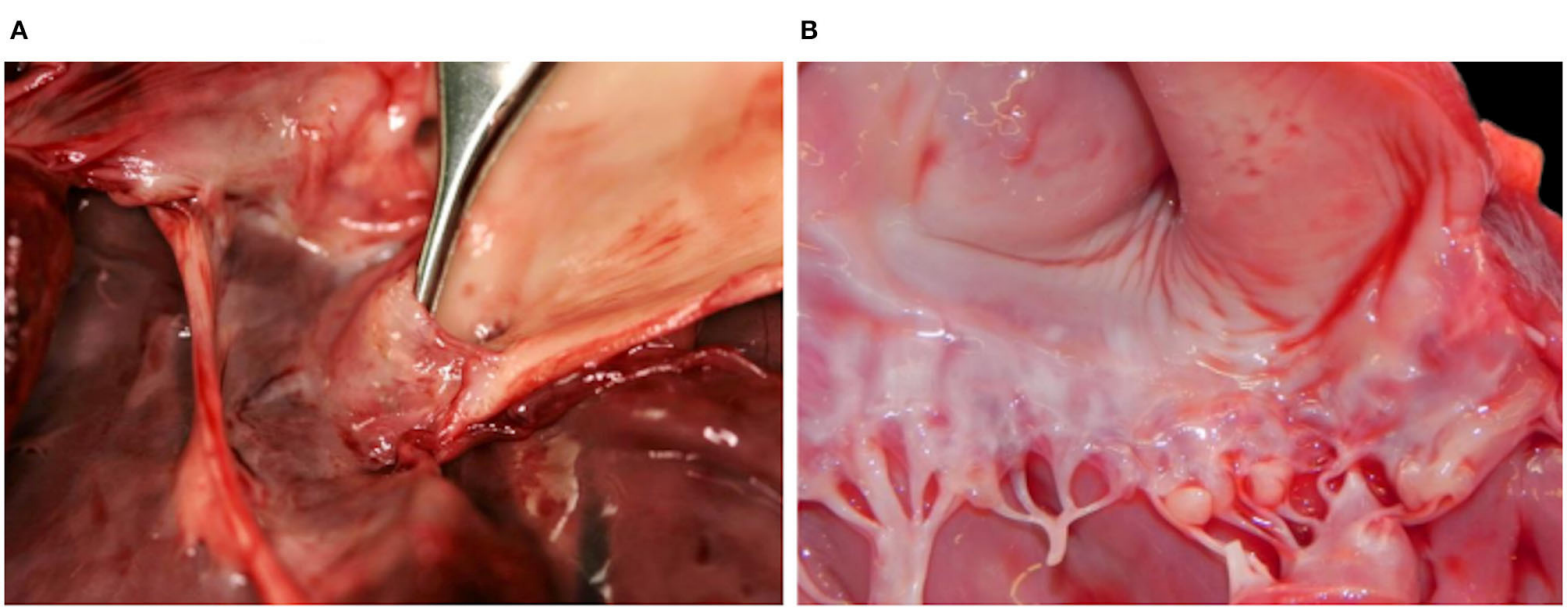
Hearts with valvular changes, Eurasian Lynx (*Lynx lynx carpathicus*); **(A)** case 3, aortic valve: stenotic fibrotic ridge expanding from, and forming a recess at the base of, the epicardium, multifocally thickened in the area comprised between the ridge and the semilunar valve (subaortic stenosis); **(B)** case 4, mitral valve: multifocal thickening and partial retraction of valvular leaflets with mild to moderate nodular thickenings of the free margins (endocardiosis).

Case 3 additionally had a subaortic stenosis with a distinct fibrotic ridge running across the left ventricle wall ~1.5 cm from the root of the aorta. A diffuse ectasia of the aortic arch was observed associated with the dilation of the left atrium and ventricle. The endocardium was thickened, particularly above the stenotic ring (Figure 3A) and in the left atrium. Endocardiosis of the mitral valve was observed in case 4 (Figure 3B) and 6.

**Figure 4:**
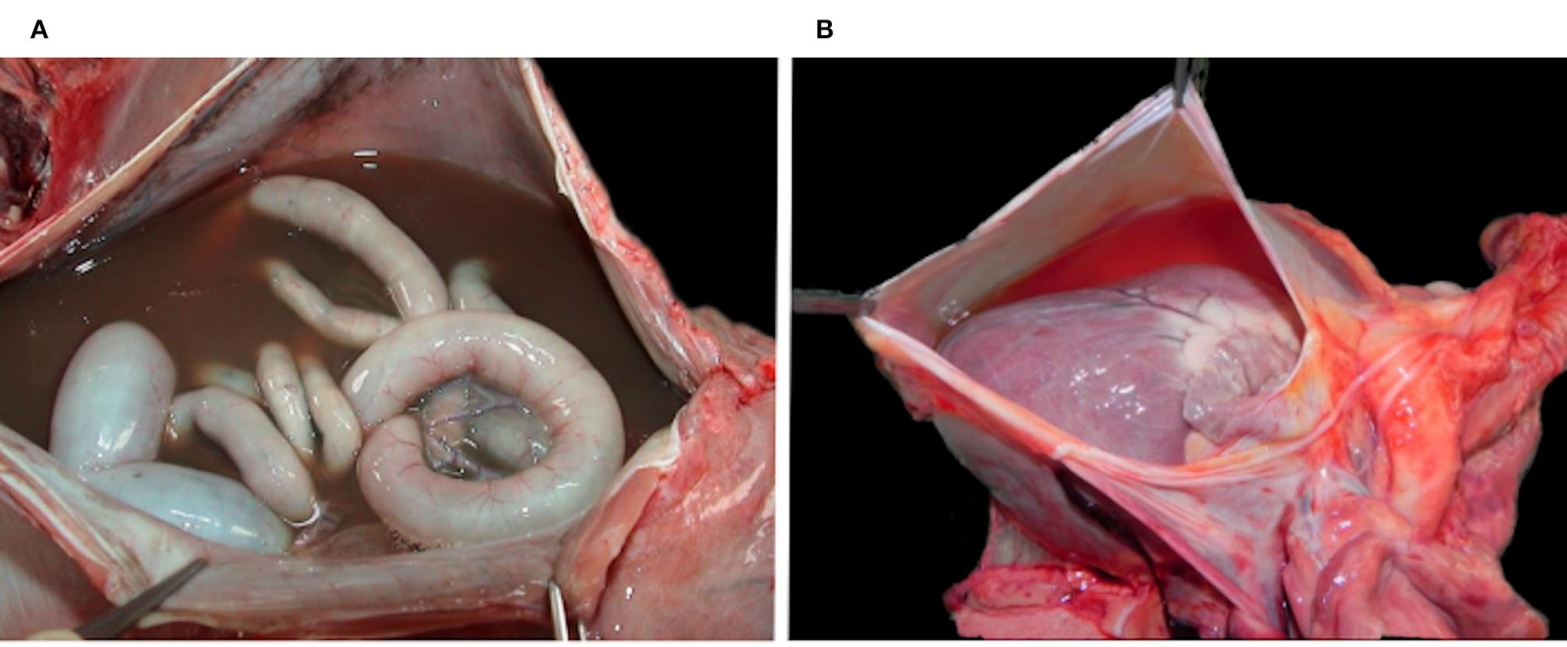
Multisystemic effusions, Eurasian lynx (*Lynx lynx carpathicus*); **(A)** case 3, abdominal cavity with severe serosanguinous effusion; **(B)** case 4, pericardial sac filled with a moderate amount of clear orange fluid.

**Figure 5:**
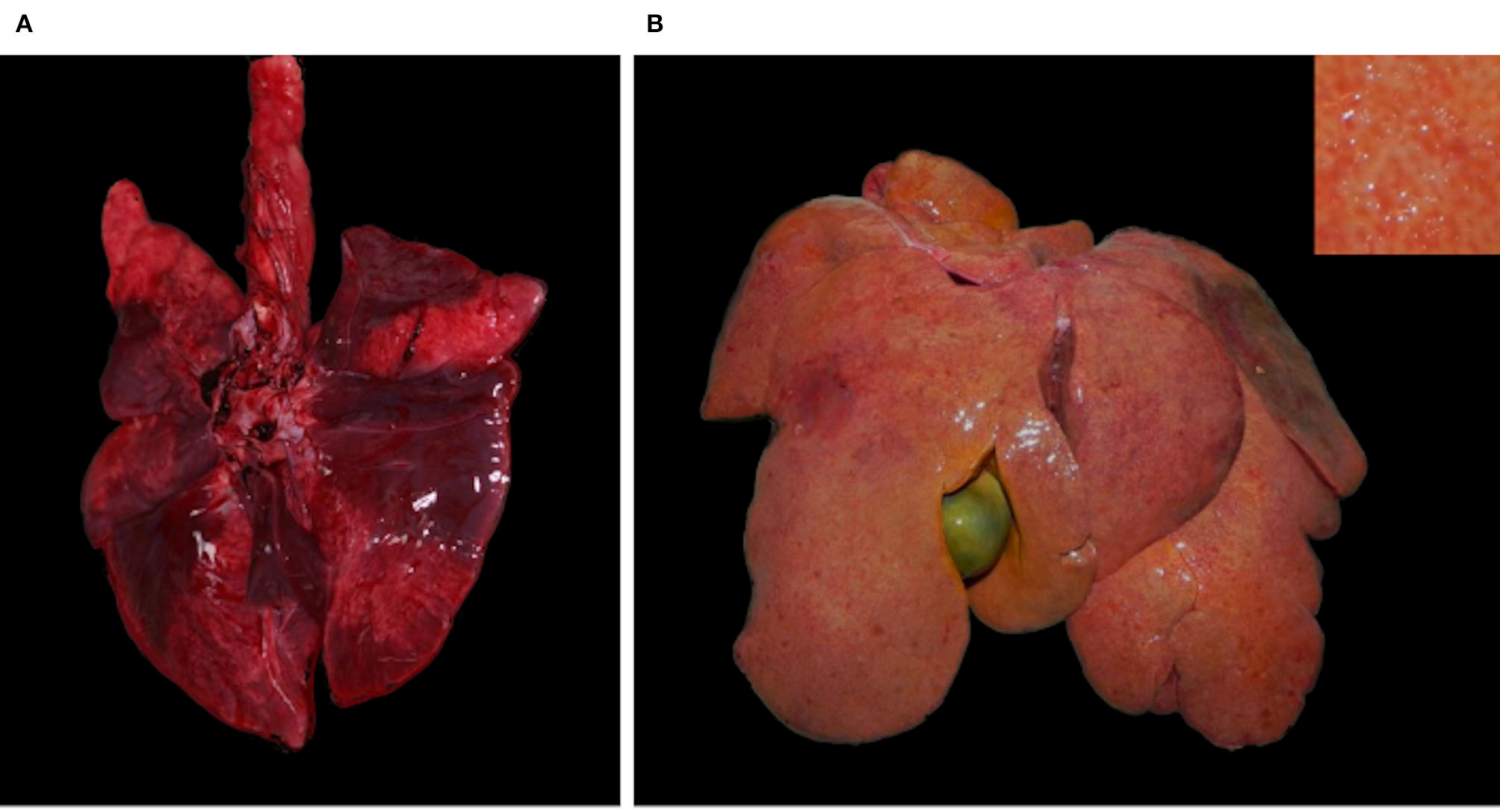
Extra-cardiac lesions, Eurasian lynx (*Lynx lynx carpathicus*); **(A)** case 3, lung; multifocal to coalescent large red foci (atelectasis) and ≪wet-like≫ appearance (edema); **(B)** case 4, liver; prominent lobular pattern (inset) with diffuse orange to yellow coloration of the parenchyma.

Case 2–5 showed a decreased body condition, ranging from moderately emaciated (reduced amount but presence of intrabdominal and coronary fat) up to cachectic (absence of coronary fat or serous fat atrophy, together with muscle atrophy), and they all had an empty stomach indicating the absence of recent food intake. All four presented with changes consistent with a right-sided congestive heart failure, including large amounts of serosanguinous fluid with fibrin deposits in the abdominal cavity (case 2, 3, and 4; Figure 4A) and severe diffuse subcutaneous edema (mainly on the caudo-ventral aspects of the body, case 2 and 3), with additional diffuse vascular congestion in case 3. There was also serous effusion in the thoracic cavity (case 2 and 3), and clear light yellow pericardial effusion (case 3, 4, and 5; Figure 4B). In case 3–5, the lung showed multifocal to coalescent large foci of atelectasis (Figure 5A), together with mild edema in the two lynx with bilateral ventricular dilation (cases 3 and 5). Additionally, the liver of case 3–5 had a prominent lobular pattern with increased consistency, along with multifocal darker (case 3) or diffuse yellow-tan (case 4) coloration (Figure 5B). Kidneys were congested in two lynx (case 3 and 4) but pale in one (case 5). Case 2 and 4 had pale mucous membranes suggestive of anemia.

The heart of case 6, an animal in excellent body condition, had an ovoid shape with bilateral ventricular hypertrophy (Figure 2) together with the valvular endocardiosis mentioned above. Additionally, case 5 showed the outcome of a previous trauma (healed rib fracture and chronic diaphragmatic hernia paramedial to the hiatus oesophagicus, with displacement of 20 cm of the omentum major and two thirds of the spleen into the thorax).

**Figure 6:**
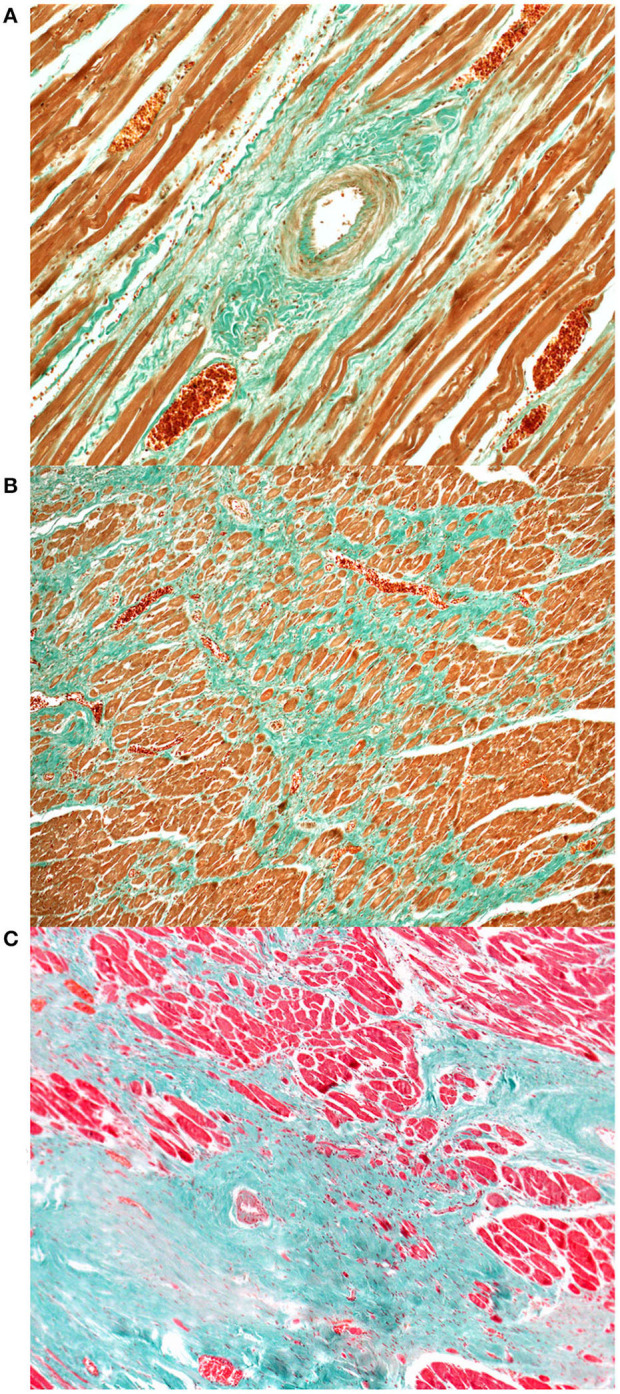
Heart, Eurasian lynx (*Lynx lynx carpathicus*), myocardial fibrosis, Masson's trichrome stain **(A)** Mild to moderate increase of the perivascular collagen along with mild to moderate intimal thickening and mild multifocal deposition of collagen between the myofibers; **(B)** Exuberant collagen is forming a dense web, entrapping, compressing and replacing several myofibers; **(C)** Islands of collagen bridging from the perivascular areas to the interstitium and replacing large numbers of myofibers.

**Figure 7:**
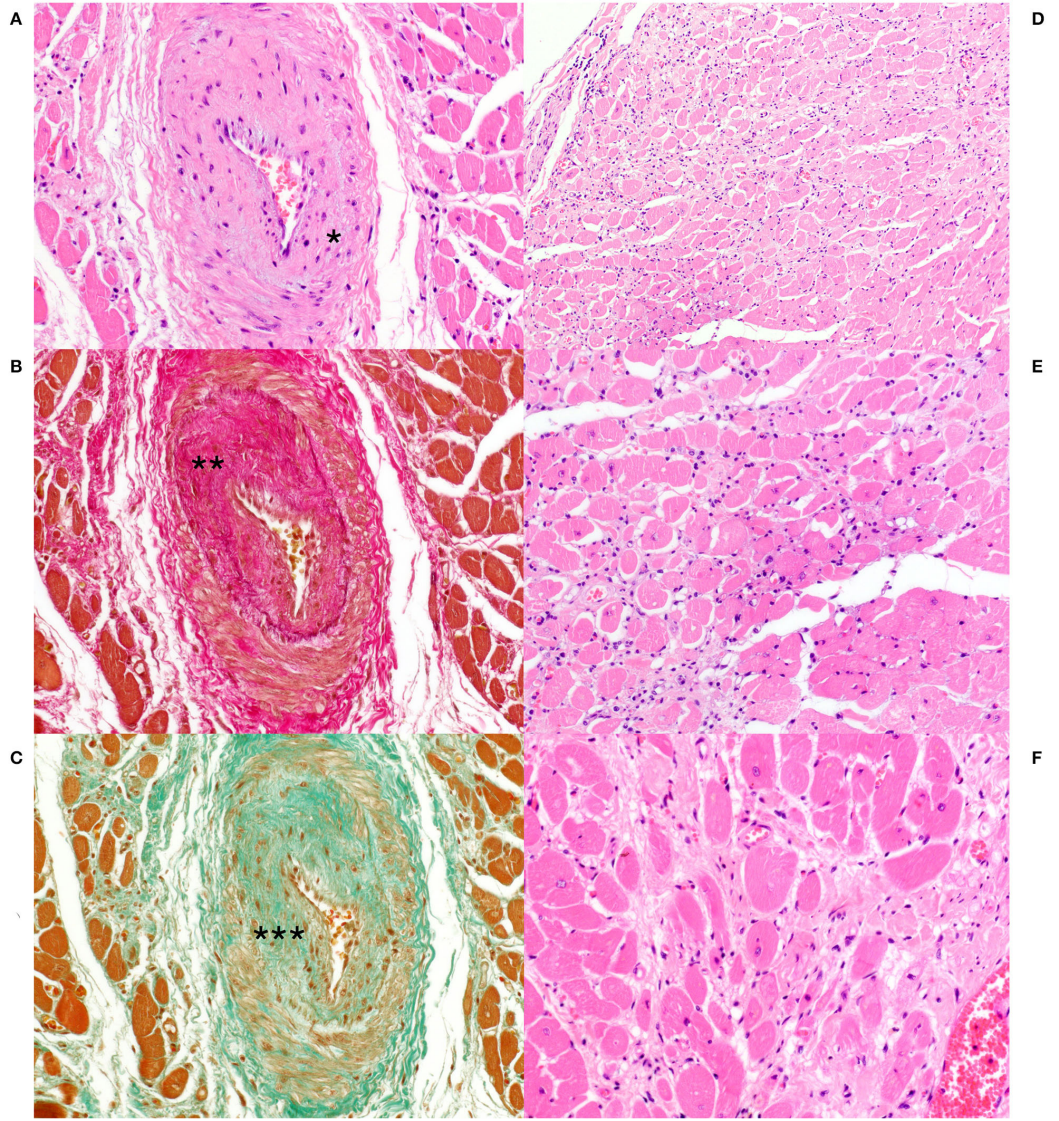
Heart, Eurasian lynx (*Lynx lynx carpathicus*); **(A)** case 3, H&E stain: Marked thickening of the arterial wall with reduction of the vascular lumen. Multifocal, mild amount of mucin-like material is observed within the intima and at the interface between the Tunica media and adventitia (Asterisk); **(B)** case 3, Van Gieson stain: marked intimal thickening secondary to deposition of collagen fibers (Asterisks) with segmental loss of the internal elastic lamina; **(C)** Case 3, Masson's trichrome stain: Presence of several myointimal cells within the thickened intimal layer (Asterisks); **(D)** case 3, H&E stain: multifocal interstitial fibrosis with reduction and replacement of the myofibers; **(E)** case 3 and **(F)** case 4, H&E stain: Myocytes with marked pleomorphism (i.e., variable morphology) and variable compression and distortion by the surrounding connective tissue. Occasional vacuolization of the sarcoplasm along with clear spaces rimming the myocyte profile. Mild to moderate hypercellularity in the interstitium.

**Figure 8:**
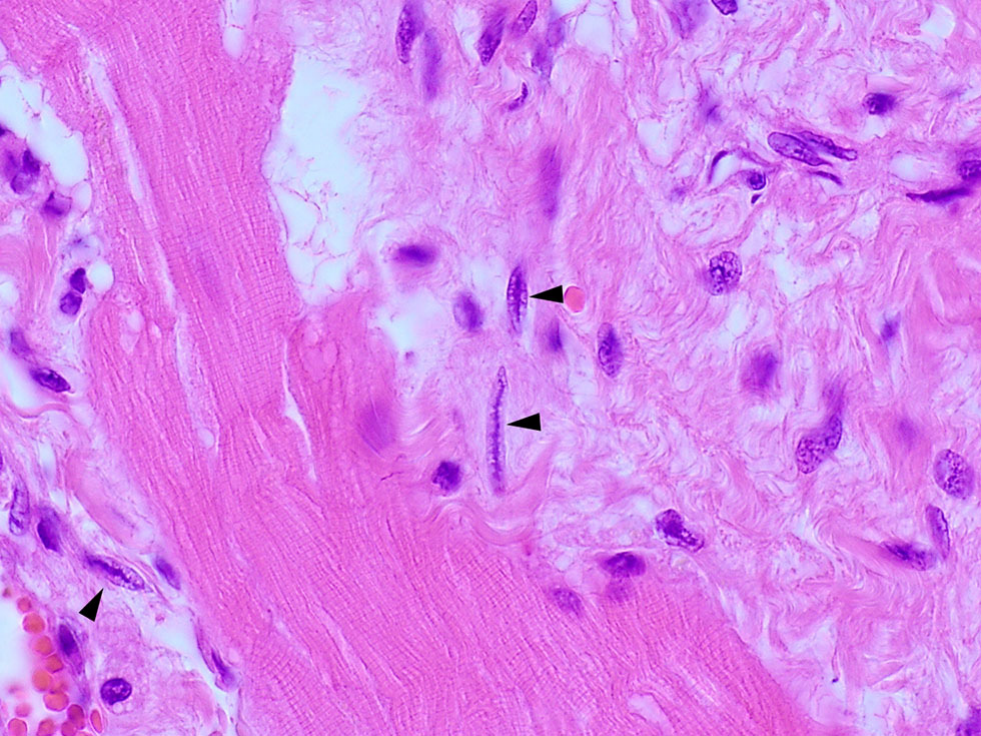
Heart, Eurasian lynx (*Lynx lynx carpathicus*), H&E stain: Multiple Anitschkow cells with their characteristic chromatin arrangement (black arrowheads).

**Figure 9:**
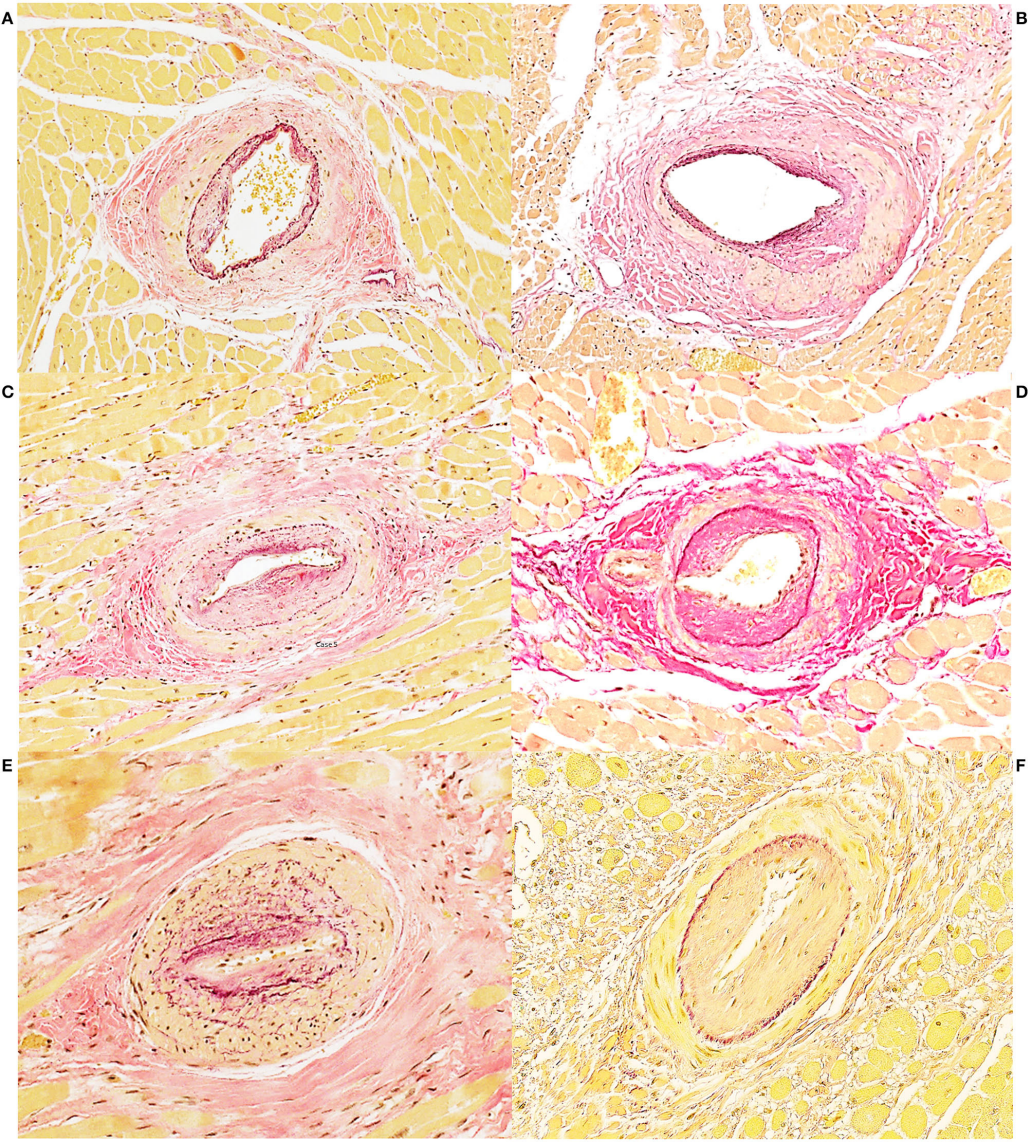
Heart, Eurasian lynx (*Lynx lynx carpathicus*), coronary arteriosclerotic lesions, Van Gieson stain **(A)** case 5: Asymmetrical, mild to moderate thickening of the media with multifocal variable accumulation of myointimal cells and reduplication of the internal elastic lamina. Multifocal mild to moderate increase of collagen fibers in the media; **(B)** case 3: Asymmetrical moderate thickening of the media with abundant collagen deposition and segmental reduplication of the internal elastic lamina. **(C)** Case 5: Moderate to severe intimal thickening with moderate proliferation of the myointimal cells, partial reduplication and fragmentation of the internal elastic lamina and lumen reduction. **(D)** case 3: Severe intimal thickening with abundant collagen deposition. Moderate increase of collagen also within the media. **(E)** case 5: Severe luminal reduction with intimal and medial thickening. Exuberant internal elastic lamina reduplication and fragmentation **(F)** case 4: Severe intimal thickening with luminal reduction and moderate myointimal cells proliferation.

Histological examination of the heart revealed similar lesions in all six lynx, consisting in mild to severe, multifocal, interstitial, perivascular and replacement fibrosis (Figures 6, 7) and Anitschkow cells (Figure 8) in the myocardium along with multifocal myocyte degeneration and loss. Anitschkow cells were present mainly in the fibrotic areas of the myocardium and in the perivascular areas of affected vessels. There was also moderate to severe multifocal arteriosclerosis with associated luminal stenosis of the small and medium-sized intramural coronary arteries (Figures 7, 9) in all cases. Myointimal cell migration into the adventitia together with fragmentation and duplication of the internal elastic lamina was common in the affected tissues (Figures 7, 9). Table 3 presents the cardiac histological changes of the six cases. Table 4 summarizes the results of the semi-quantitative evaluation of myocardial fibrosis and arteriosclerosis. In addition, case 3 showed a neutrophilic epicarditis with edema and multifocal mild myocyte multinucleation. In contrast to the others, interstitial fibrosis was more severe in the right ventricle of this lynx and it was associated with myocyte degeneration. In case 4, there was a moderate myocyte degeneration characterized by sarcoplasma vacuolization, prominent anysocytosis and a mild lymphocytic infiltration. In case 6, mucin-like material embedding variable-extensive collagen nodules and elastic fibers were found mainly in the distal part of the mitral leaflet together with hemosiderin-laden macrophages (endocardiosis). Similar material was also occasionally observed in between the collagen fibers of the tunica adventitia.

**Table 3:** Assessment and scoring of the main histopathological features of the heart of six free-ranging Eurasian lynx (subspecies *Lynx lynx carpathicus*) from Switzerland affected by cardiomyopathy.

**Lynx case Nr**.	**Vascular**	**Interstitial**	**Myocytes**
1	n/a	n/a	n/a
2	AS 1	MF 1 Minimal inflammation Anitschkow cells 1	Degeneration & loss Occasional waving
3	AS 2	MF 2 Minimal inflammation Anitschkow cells 3	Degeneration & loss multifocal mild myocyte polyploidy
4	AS 1	MF 2 Minimal inflammation Anitschkow cells 1	Degeneration & loss Mild waving
5	AS 3	MF 2-3 Mild inflammation Anitschkow cells 1	Degeneration & loss
6	AS 2-3	MF 2 Minimal inflammation Anitschkow cells 1	Extensive degeneration and loss

**Table 4:** Post-mortem heart findings in six Eurasian lynx (subspecies *Lynx lynx carpathicus*) from Switzerland affected by cardiomyopathy and a healthy individual (control) for comparison.

**Parameters**		**Case 1**	**Case 2**	**Case 3**	**Case 4**	**Case 5**	**Case 6**	**Control**
Clinical findings	Heart murmur	n/a	n/a	Yes	Yes	Yes	n/a	None
Gross changes	Cardiomegaly	None	RV dilation	LV and RV dilation	LV dilation	LV and RV dilation	LV and RV hypertrophy	None
	Myocardial fibrosis	None	None	Severe	Severe	Severe	Severe	None
	Valvular changes	Mitral EC	None	Subaortic stenosis	Mitral EC	None	Mitral EC	None
Morphometrics	Heart weight (g)	n/a	n/a	130.0	145.5	101.5	129.0	94.0
	Relative HW (%)^a^	n/a	n/a	0.78	0.90	0.66	0.52	0.50
	RV:S:LV^b^	n/a	n/a	3:10:5	4:10:7	3:10:8	7:16:20	4:11:13
	RV:S:LV ratio	n/a	n/a	1:3.3:1.7	1:1.7:1.4	1:3.3:2.7	1:2.3:2.9	1:2.8:3.3
	RV: LV ratio	n/a	n/a	0.6	0.57	0.38	0.35	0.31
Histology	MF+AS score^c^ (total)	2 + 3 (5)^d^	1 + 1 (2)	2 + 2 (4)	1 + 2 (3)	3 + 2.5 (5.5)	2.5 + 2 (4.5)	0 + 0 (0)

*EC,endocardiosis; n/a, not applicable*.

a *Heart weight (HW) / Body weight (BW) in grams x100. The mean ratio HW:BW obtained for apparently healthy Eurasian lynx from Switzerland, based on 10 adult males in good body condition, was 0.51 (range: 0.45–0.65). The ratio obtained for two cachectic adult males without evidence of clinical cardiac disease was 0.64 and 0.69, respectively. The HW:BW ratio in healthy Northern lynx was within the same range as for domestic cats (42), i.e., 0.58 ± 0.28–0.88 (13)*.

b *Thickness of the right ventricle free wall (RV), interventricular septum (S) and left ventricle free wall (LV) measured in millimeters. For comparison, the mean RV:S:LV ratio for 11 adult male Eurasian lynx without evidence of clinical cardiac disease was 5:13:15 mm (or 1:2.6:3 ratio, i.e., close to the 1:3:3 ratio in healthy domestic cats (16)*.

c *Summary of the semi-quantitative evaluation of myocardial fibrosis (MF) and coronary arteriosclerosis (AS) shown in Table 3*.

d *Scores based on the pathology report descriptions (no archived histological slides or paraffin blocks)*.

Under light microscopy, the lungs revealed the presence of diffuse edema (interstitial, alveolar, peribronchial and/or perivascular) and multifocal atelectasis of varying severity in four lynx each (case 1–3, 5, and case 2–5, respectively). Furthermore, heart-failure cells were seen in two (case 2 and 3). Additional findings included a subpleural hemangioma (case 2), multifocal emphysema (case 1 and 5), mild to moderate hemorrhages (cases 2 and 6), multifocal mild calcifications (case 4 and 6), and a mild multifocal histiocytic to lymphocytic interstitial pneumonia (case 4).

Microscopic liver changes were observed in four lynx (case 2–5). Three animals showed Ito-cells hyperplasia (case 2–4), with additional moderate to severe hemosiderosis in case 2 and 3, which were both found dead in a status of cachexia. Furthermore, the liver of case 3 was characterized by a severe centrolobular to panlobular fibrosis, together with multifocal moderate arteriosclerosis and mild cholestasis. In case 4, there was moderate centrolobular degeneration and mild centrolobular fibrosis together with mild neutrophilic and lymphocytic infiltrates, mild hemosiderosis, cholestasis and sinusoidal dilation.

Additional findings were acute necrotizing pancreatitis with fibrinous and plasmacellular peripancreatitis (case 2), peritonitis (case 2 and 3), interstitial edema in the hind leg muscles (case 3), mild to moderate lymphoid follicle hyperplasia in the mesenterial lymph nodes (case 2), spleen lymphoid depletion (case 3 and 4), mild membranous glomerulopathy (case 3, 5, and 6), and mild multifocal lymphoplasmacytic meningitis (case 6).

A comparative summary of the main pathological findings, data on heart morphology and scoring of histological heart lesions are presented in Tables 4, 5.

**Table 5:** Post-mortem findings in other organs than the heart in six Eurasian lynx (subspecies *Lynx lynx carpathicus*) from Switzerland affected by cardiomyopathy.

**Parameters**	**Case 1**	**Case 2**	**Case 3**	**Case 4**	**Case 5**	**Case 6**
Subcutaneous edema	None	Severe	Severe	None	None	None
Abdominal effusion	None	Severe	Severe	Severe	None	None
Thoracic effusion	None	Moderate	Moderate	None	None	None
Pericardial effusion	None	None	Mild-moderate	Moderate-severe	Mild	None
Lung edema	Severe	Mild	Mild	None	Moderate	None
Lung atelectasis	None	Mild	Moderate-severe	Moderate-severe	Moderate-severe	None
Liver changes^a^	None	Mild	Severe	Mild	Mild	None
Kidney changes^b^	None	None	Mild	Mild	Mild	Mild

a *Prominent lobular pattern and increased consistency (case 2–5); altered liver color (multifocally darker in case 3, and diffuse yellow-tan in case 4); mild to severe hemosiderosis (case 2–4) and sinusoidal dilation in case 4; Ito-cell activation (case 2–4); mild cholestasis as well as multifocal, moderate arteriosclerosis (case 3); fibrosis (severe and centrolobular to panlobular in case 3, mild and centrolobular in case 4); moderate centrolobular hepatocyte degeneration as well as mild neutrophilic and lymphocytic inflammation (case 4)*.

b *Mild congestion (case 3 and 4); mild color alteration (pale, case 5); mild membranous glomerulopathy (case 3, 5, 6)*.

## Discussion

This study reveals the existence of previously undescribed cardiac disorders in Eurasian lynx from a reintroduced population in Switzerland. The pathological features consistent throughout the cases were the presence of myocardial fibrosis with associated Anitschkow cells, and coronary arteriosclerosis with intimal proliferation, which were pronounced in all lynx except case 2. Despite inter-individual differences, shared pathological features suggest that the six lynx were affected by the same disease entity.

While case 1 unexpectedly died during anesthesia and presented with a severe acute lung edema, case 2–4 had macroscopic pathological changes characteristics of a congestive right-sided heart failure. The most advanced disease picture, including liver fibrosis and congestion, was observed in case 3, the animal with a subaortic stenosis, which had presented with a heart murmur at clinical examination and died spontaneously. Case 4, which was even older but had been culled due to disease signs, seemed to be in an intermediary position between case 2 and 3 in term of disease severity and chronicity. In line with the clinicopathological features, relative heart weight and both heart wall ratios of case 3 and 4 (no available morphologic heart data for case 2) strongly differed from those of the control animal. Case 5 (euthanized) and 6 (hit by car) had major macroscopic and histological cardiac lesions although they died prematurely, but they presented with fewer (case 5) and no (case 6) major systemic changes. Accordingly, case 5 may have been in an earlier disease stage than case 2–4, and disease may have been subclinical in case 6. In these two last cases, relative heart weight and right/left ventricular wall ratios were similar to those of the control but they had bilateral ventricular alterations (dilation and hypertrophy, respectively), possibly explaining that the wall ratios did not diverge much from the control ratio. In case 6, all three walls were over the average values of adult males without evidence of cardiac disease, in agreement with a bilateral ventricular hypertrophy, although the similar relative heart weight was not consistent with an increased heart mass. It is conceivable that this animal with hypertrophic heart features may have been at an earlier disease stage than cases 2–5, which showed heart dilation. In animal models, concentric myocardial hypertrophy and fibrosis precede dilation, which marks progression toward decompensated heart failure and systolic dysfunction (47).

Scores of the histological main lesions were highest in case 5, followed by case 1 (unexpected death, not assessed by the authors), 6, 3, and 4, and they were lowest in case 2, not paralleling the clinicopathological findings. It is known that there is not necessarily full correspondence between the severity of gross and microscopic abnormalities within the heart and the presence of clinical signs attributable to them. While major structural abnormalities may be found incidentally after death, there may be relatively mild but critically located lesions resulting in marked functional disturbance (48). Sudden cardiac death may occur when the coronary perfusion is impaired (49) or as a result of ventricular arrhythmias, e.g., in case of (mainly interstitial) myocardial fibrosis (50). Unexpected death frequently occur in both animals and humans with subclinical heart disease (7, 16, 43), meaning that heart disease may be evident only postmortem (13).

Capture procedure and anesthesia likely were stressing factors in case 1. Anesthesia is a procedure associated with an increased risk in cardiac patients, whether they present with clinical signs of heart failure or not. Anesthesia was performed using an alpha-2 adrenergic drug, a category of sedatives that is considered as contra-indicated in patients with cardiac disease (51). Alpha2-adrenoceptor agonist and ketamine combinations provide a rapid reversible anesthesia but may cause severe sustained hypertension, putting animals at risk for development of, among others, pulmonary edema and cardiac failure (52). Therefore, although case 3 survived the anesthesia despite an advanced stage of heart failure, subclinical cardiac disease likely predisposed case 1 to a fatal capture accident. The anesthetic drugs may also have contributed to the respiratory insufficiency and lung edema observed in case 5.

The three lynx with chronic heart disease had cardiomegaly matching the criteria of an eccentric hypertrophy, considering that the cardiac volume and mass were increased, although the ventricular free walls were of normal to decreased thickness and the papillary muscles were attenuated (13, 49). However, the lack of congestion in the stomach, intestine, spleen and liver (except for case 3), and the presence of liver fibrosis suggest a more chronic course in these lynx than typically observed in domestic animals with congestive heart disease (13). While the last two cases were older males, case 2 was a young female in the late growth period. She presented only with a right heart dilation and, in agreement with her younger age, the apparent absence of liver involvement pointed at a faster disease course.

Case 1, 3, 4, and 6 had valvular disease additionally to the lesions in the myocardium and coronary arteries. Subvalvular aortic stenosis and mitral endocardiosis are common in dogs but not in cats. They are typically associated with a left-sided cardiac dysfunction that may end in a congestive heart failure characterized by interstitial and alveolar pulmonary edema. Myocardial fibrosis may develop with both valvular pathologies and may be associated with arteriosclerosis. However, animals with aortic stenosis are mainly prone to sudden death, and endocardiosis is a frequent incidental finding at necropsy (13, 49). While the observed dilation of the aortic arch of case 3 fitted to a post-stenotic dilation of the aorta, the dilation of the left atrium and ventricle did not fit with the compensatory concentric hypertrophy (thickening of the ventricular walls) expected in presence of a subaortic stenosis (13, 49). Differently, dilation of the left ventricle in case 4 was consistent with the eccentric hypertrophy (ventricular dilation) expected in atrio-ventricular valve disease (13). Nevertheless, according to the nearly absent signs of a left-sided heart failure in the investigated lynx, valvular abnormalities were considered exacerbating factors in lynx 3 and 4 but not primary etiologies. Since aortic stenoses are a cause of heart murmur (53), the observed subvalvular stenosis in case 3 may explain the murmur heard at capture. However, this is not supported by the findings of case 5, who had a murmur but inconspicuous valves. Myocardial diseases alone are indeed associated with murmurs (54–56).

All lynx had varying degrees of fibrosis in the myocardium. Myocardial fibrosis is associated with increased ventricular stiffness, which compromises ventricular systolic (and diastolic) function, abnormal cardiac remodeling and development of ventricular dilation, and activation of compensatory mechanisms that increase sodium and water retention to increase venous return to the heart (47, 49, 57, 58). Eccentric myocardial hypertrophy occurs as a compensatory mechanism to allow the ventricle to pump a relatively normal amount of blood despite abnormal systolic function (49). In humans, cardiac fibrosis is a strong predictor of adverse outcome (57). Therefore, the observed myocardial lesions are consistent with the observed disease in the lynx described here and the fatal outcome in case 3.

The origin of the observed myocardial lesions is unknown. In animals and man, fibrosis is a common feature of most myocardial diseases. The adult mammalian heart has negligible regenerative capacity, and once myocytes are lost, there is progressive scavenging of the necrotic remnants and replacement by fibrosis, mainly by activation of resident interstitial myofibroblasts, a process that aims at preserving the structural integrity of the heart. Pathophysiological mechanisms leading to fibrosis development are various, some being acute as in myocardial infarction, others being progressive. Accordingly, not only cardiomyocyte death but also injurious stimuli such as pressure and/or volume overload, metabolic dysfunction, myocardial inflammation, as well as aging, may cause interstitial and perivascular fibrosis in the absence of infarction (57). However, the lynx cases included young individuals still in the growth phase, and no concurrent disease susceptible to explain the cardiac lesions were detected at necropsy. Interstitial cardiac fibrosis has been associated with the delayed peracute syndrome of capture myopathy described in animals that have experienced an earlier capture event. However, re-captured animals affected by this syndrome die within a few minutes. Furthermore, felids do not belong to the mammals prone to develop capture myopathy (59), and two of six cases (case 2 and 6) had never experienced a capture, while the control had been anesthetized multiple times.

Another feature of the lynx cardiomyopathy was coronary arteriosclerosis. Arteriosclerosis, or chronic arterial stenosis, has been described in multiple animal species including dogs and cats, swine, bovine, monkeys and mice (60, 61). It usually results from proliferative and degenerative changes and is common in domestic animals, with age-related increasing frequency and severity. Despite being often an incidental, clinically irrelevant finding, it is frequently associated with heart diseases, e.g., in dogs with mitral valve disorders and cats with HCM, cats and dogs with *Chlamydia* sp. infection, and dogs experiencing sudden death (60).

Although arteriosclerosis and myocardial fibrosis can occur independently from each other (60), myocardial infarction and coronary arteriosclerosis are principal causes of heart failure in humans, and there is increasing evidence of an association of myocardial fibrosis and arterial lesions in both animals and humans, with fibrosis believed to result from ischemia caused by arterial stenosis (60–65). Accordingly, arteriosclerosis may have been the cause of myocardial injury and replacement fibrosis in the lynx.

Following pathological examination of case 3, we conducted a pilot study on the histopathology of Swiss lynx hearts using archived H&E slides. We compared them with a small number of H&E slides from Swedish lynx (Northern lynx) and found a significant difference in prevalence of combined myocardial fibrosis and arteriosclerosis (65.8 and 23.8%, respectively), which affected mainly adult males (66). In a second phase of this pilot study we used a larger sample size from both countries, processed all samples in the same laboratory and used VG instead of H&E stain. In this second sample set, the mentioned histological anomalies occurred at a prevalence close to 70% in both countries while moderate and severe lesions were most common in the Swiss Alps, suggesting that mild arteriosclerosis and myocard fibrosis are common in Eurasian lynx but supporting the data of the first study phase as concerns the difference between Sweden and Switzerland. Furthermore, the differences with the first phase of our study demonstrated the importance of harmonized methods to conduct proper comparisons (67). Interestingly, a subsequent study in lynx from Finland underlined the existence of population-related differences even more, as minimal to mild fibrosis was detected in 56% of 63 Northern lynx heart but arteriosclerosis was never observed (42).

We found Anitschkow cells in all six lynx, differently from animals without evidence of cardiac disease, including the control animal. Anitschkow cells are characteristic mononuclear cells with “caterpillar” - like nuclei, which are usually observed in the myocardium and in coronary vessel walls and may represent modified myocytes. They are typically found in humans affected by rheumatic heart disease and other cardiac pathologies, and in animals of various species with myocarditis, myocardial necrosis, degenerative and inflammatory endocardial diseases, or arteriosclerosis (13, 60, 68). Accordingly, the presence of Anitschkow cells may also be linked to myocardial disorders in lynx.

Given the absence of obvious signs of infection (blood values and clinical observations, pathological findings) in all studied lynx, a genetic origin of this cardiomyopathy should be considered. Genetic factors are increasingly recognized as a cause of cardiac disorders, including cardiomyopathies with and without associated intramural coronary artery wall abnormalities (2, 4, 69). For example, genetic anomalies have been associated with HCM in cats and humans (21, 69, 70), DCM in cattle, dogs and humans (2, 4, 71, 72) and other cardiomyopathies in various species, including animal models such as mice and rhesus macaques (73). The six affected lynx came from the same population, which has undergone a severe population bottleneck and is characterized by a reduced level of heterozygocity (37, 74). Although these six cases are few compared to the mortality recorded in lynx in Switzerland (311 lynx submitted to necropsy from 1987 to 2019), diseased wild animals are less likely to be discovered compared to those that die of anthropogenic causes and are therefore underrepresented in necropsy material (45, 75). This may be particularly true for animals experiencing sudden cardiac death. The repeated occurrence of a yet undescribed disease in the reintroduced Swiss lynx population deserves attention, especially considering that genetic threats to small isolated populations are gradual processes that show long-term effects (26). As a genetic origin of the observed conditions would have serious consequences on on-going conservation programs in Europe, this hypothesis warrants further investigations.

In conclusion, the investigated lynx were affected by a myocardial disease with coronary arteriosclerosis, potentially leading either to unexpected death or to a right-sided congestive heart failure, and associated with a heart murmur in the two cases that could be examined clinically. Heart function was likely further compromised by valvular disorders in four of six cases. As the heart alterations did not match any of the three main cardiomyopathy categories (HCM, DCM, RCM), they must be regarded as a cardiomyopathy of non-specified phenotype with potential genetic background. The cases presented here span over 20 years, illustrating the value of long-term health monitoring of reintroduced wildlife populations and of performing necropsies on wild animals that die during capture events. This study also highlights the importance of veterinary investigations and standardized necropsy protocols in the framework of conservation programs.

## Data Availability Statement

The original contributions presented in the study are included in the article, further inquiries can be directed to the corresponding author/s.

## Ethics Statement

Ethical review and approval was not required for the animal study because investigations were performed on dead animals submitted to necropsy. Data previously collected on live animals were obtained in the framework of ecological studies run independently of the present study and conducted with the necessary authorizations of the Swiss cantonal ethics committees according to the Swiss law on animal experiments.

## Author Contributions

M-PR-D designed, performed the study, and wrote the manuscript. NR and FO provided pathology expertise and contributed to pathology data collection. FO described the histological pictures. M-PR-D, MP, and RM carried out clinical examinations. M-PR-D, SZ-G, and RM performed necropsies. UB initiated veterinary examinations of lynx in Switzerland, coordinated the ecological projects, and contributed to field work. AR organized the captures and radio-tracked the lynx. AK provided cardiology expertise and performed an in-depth cardiological examination of the control animal. All authors critically read and approved the manuscript.

## Conflict of Interest

The authors declare that the research was conducted in the absence of any commercial or financial relationships that could be construed as a potential conflict of interest.
